# Hepatic triglyceride accumulation via endoplasmic reticulum stress-induced SREBP-1 activation is regulated by ceramide synthases

**DOI:** 10.1038/s12276-019-0340-1

**Published:** 2019-11-01

**Authors:** Ye-Ryung Kim, Eun-Ji Lee, Kyong-Oh Shin, Min Hee Kim, Yael Pewzner-Jung, Yong-Moon Lee, Joo-Won Park, Anthony H. Futerman, Woo-Jae Park

**Affiliations:** 1 Department of Biochemistry, College of Medicine, Ewha Womans University, Seoul, 07084 Republic of Korea; 2 Department of Biochemistry, College of Medicine, Gachon University, Incheon, 21999 Republic of Korea; 3 College of Pharmacy, Chungbuk National University, Chongju, 28644 Republic of Korea; 4 Department of Biomolecular Sciences, Weizmann Institute of Science, Rehovot, 76100 Israel; 5 Department of Health Sciences and Technology, GAIHST, Gachon University, Incheon, 21999 Republic of Korea

**Keywords:** Mechanisms of disease, Metabolic disorders, Obesity

## Abstract

The endoplasmic reticulum (ER) is not only important for protein synthesis and folding but is also crucial for lipid synthesis and metabolism. In the current study, we demonstrate an important role of ceramide synthases (CerS) in ER stress and NAFLD progression. Ceramide is important in sphingolipid metabolism, and its acyl chain length is determined by a family of six CerS in mammals. CerS2 generates C22-C24 ceramides, and CerS5 or CerS6 produces C16 ceramide. To gain insight into the role of CerS in NAFLD, we used a high-fat diet (HFD)-induced NAFLD mouse model. Decreased levels of CerS2 and increased levels of CerS6 were observed in the steatotic livers of mice fed a HFD. In vitro experiments with Hep3B cells indicated the protective role of CerS2 and the detrimental role of CerS6 in the ER stress response induced by palmitate treatment. In particular, CerS6 overexpression increased sterol regulatory element-binding protein-1 (SREBP-1) cleavage with decreased levels of INSIG-1, leading to increased lipogenesis. Blocking ER stress abrogated the detrimental effects of CerS6 on palmitate-induced SREBP-1 cleavage. In accordance with the protective role of CerS2 in the palmitate-induced ER stress response, CerS2 knockdown enhanced ER stress and SREBP-1 cleavage, and CerS2 heterozygote livers exhibited a stronger ER stress response and higher triglyceride levels following HFD. Finally, treatment with a low dose of bortezomib increased hepatic CerS2 expression and protected the development of NAFLD following HFD. These results indicate that CerS and its derivatives impact hepatic ER stress and lipogenesis differently and might be therapeutic targets for NAFLD.

## Liver disease: Boosting a protective protein

Promoting the activity of a protective membrane protein may help limit the development of non-alcoholic fatty liver disease (NAFLD) in obesity. Stress on a key cellular organelle, the endoplasmic reticulum (ER), contributes to NAFLD progression. Woo-Jae Park at Gachon University in Incheon, Joo-Won Park at Ewha Womans University, Seoul, and co-workers across South Korea have uncovered the role of a family of ER membrane proteins called ceramide synthases (CerS) in the regulation of ER stress during disease development. The team found increased levels of CerS6 in the livers of mouse fed a high-fat diet, while CerS2 decreased. The increased C16-ceramide by CerS6 overexpression triggered excess fat formation by increasing ER stress and SREBP-1 cleavage. However, when the team enhanced the expression of CerS2 using an existing chemotherapy drug, mice were protected from developing NAFLD.

## Introduction

Nonalcoholic fatty liver disease (NAFLD), a major liver disease worldwide, is associated with insulin resistance and frequently displays features of metabolic syndrome^1,2^. According to a histological spectrum, NAFLD ranges from simple steatosis (accumulation of hepatic fat) to nonalcoholic steatohepatitis, which is associated with an increased risk for progression to cirrhosis^3,4^. The activation of sterol regulatory element-binding protein-1c (SREBP-1c), a transcriptional activator of lipogenic enzymes such as stearoyl coenzyme-A desaturase1 (SCD1) and fatty acid synthase (FAS), plays an important role in the pathogenesis of NAFLD via an increased rate of fatty acid synthesis^5^. The overexpression of SREBP-1c results in increased lipogenesis and classic fatty liver^6^; in contrast, the inactivation of the SREBP-1 gene in ob/ob mice leads to a 50% reduction in triglycerides^7^.

Owing to the obesity epidemic, the incidence of NAFLD has been increasing dramatically, and endoplasmic reticulum (ER) stress, which occurs when the folding capability of the ER fails to accommodate the load of unfolded proteins^1^, has been shown to contribute to the development and progression of NAFLD under obese conditions^1^. In response to ER stress, the unfolded protein response is executed as an adaptive mechanism to restore homeostasis in the ER and is mediated by ER-located transmembrane proteins, inositol-requiring protein-1, protein kinase RNA-like ER kinase (PERK), and activating transcription factor-6^1^. Glucose-regulated protein of 78 kDa (GRP78), which is normally associated with the PERK monomer, is dissociated from PERK upon ER stress, and PERK is activated and phosphorylates eukaryotic initiation factor 2 α (eIF2α) at ser-51, resulting in the suppression of global protein translation and a reduced protein load in the ER^1^. PERK also mediates ER stress-induced cell death by inducing the proapoptotic transcription factor CCAAT-enhancer-binding protein homologous protein (CHOP)^1^. Several studies have reported the crucial role of ER stress in the pathogenesis of NAFLD. For example, GRP78 overexpression in the livers of ob/ob mice reduced ER stress and inhibited SREBP-1c cleavage, resulting in decreased hepatic triglyceride and cholesterol levels^8^. In addition, livers deficient in activating transcription factor 6α or inositol-requiring enzyme 1α display higher ER stress and more severe steatosis upon acute ER stress or upon the administration of a high-fat diet (HFD)^9,10^.

Ceramides are lipid species that exert various biological effects, such as cellular proliferation, differentiation, and cell death and have been implicated in several pathways involved in insulin resistance, oxidative stress, and inflammation, all of which are linked to NAFLD^3^. Ceramide can be generated either de novo by the *N*-acylation of sphenoid long-chain bases or via the degradation of sphingolipids (SLs), such as sphingomyelin and glycosphingolipids^11,12^. Ceramide synthases (CerS) determine the specific acyl chain lengths of ceramides, and mammals have six CerS^11,12^. More specifically, CerS1 and CerS2 primarily generate C18 ceramide^13^ and C22-C24 ceramides^14^, respectively. CerS3 produces C26-C34 ceramides^15^, whereas CerS4 generates C18-C20 ceramides^16^. Both CerS5 and CerS6 share acyl chain length specificity, generating C16 ceramide^17,18^. The different roles of ceramide depending on acyl chain length have been confirmed by the distinct phenotypes of various CerS-deficient mice generated over the past few years^12^. For example, decreased C16 ceramide levels in CerS5- or CerS6-deficient mice resulted in improved glucose homeostasis^19,20^; however, decreased C22-C24 ceramides in CerS2-null mice induced insulin resistance^21,22^. Although ceramides have been implicated in several pathways linked to NAFLD^3,23^, the precise role of ceramides with distinct acyl chain lengths in NAFLD is not fully understood.

The present study proposes a mechanism through which ceramides contribute to the development and progression of NAFLD via regulating ER stress and SREBP-1 activation. We also demonstrate a protective role of low-dose bortezomib in the development of NAFLD by mediating an increase in CerS2.

## Materials and methods

### Materials

Agents were purchased/obtained as follows: (1) bortezomib (Biovision, Palo Alto, CA); (2) fumonisin B1, palmitate, 4-PBA (4-phenylbutyric acid), TUDCA (tauroursodeoxycholic acid), and anti-α-tubulin, anti-HA and anti-CerS2 antibodies (Sigma-Aldrich, St Louis, MO); (3) ceramides (fatty acyl lengths C16, C18, C20, C22, and C24) and C17 ceramide (d_17:1_/C_18:0_) (Avanti Polar Lipid, Alabaster, AL); (4) anti-GRP78, anti-CHOP, anti-FAS, anti-phospho-eiF2α, anti-eiF2α, anti-PERK, and anti-SCD-1 antibodies (Cell Signaling Technology, Beverly, MA); (5) anti-SREBP-1, anti-phospho-PERK and anti-CerS6 antibodies (Santa Cruz Biotechnology, Santa Cruz, CA); (6) anti-GAPDH (glyceraldehyde 3-phosphate dehydrogenase) antibody (Millipore, Temecula, CA); (7) anti-INSIG-1 antibody (Abcam, Cambridge, MA); (8) anti-mouse-HRP (horseradish peroxidase) and anti-rabbit-HRP antibodies (Jackson Laboratory, Bar Harbor, ME).

### Animal experiments

The experimental procedures were approved by the Animal Ethics Committee at Ewha Womans University College of Medicine (ESM#14-0280), and all experiments were carried out in accordance with the approved guidelines and regulations. All mice were housed under specific pathogen-free conditions on a 12 h light/dark cycle with free access to food and water. Total hepatic ceramide level has been reported to be increased with age^24^. To examine hepatic protein expression and ceramide levels at different time points (0, 6, 12, 18, and 24 weeks) during high-fat diet (HFD) administration, C57BL/6 mice (male, 16–18 g, 6 weeks old) were purchased from Orient Bio Inc. (Seongnam-si, South Korea) and started on the HFD (D12492, 60% fat, 20% carbohydrate, 20% protein, 5.24 kcal/g; Research Diets Inc., New Brunswick, NJ) for different lengths of time and then killed at the same time. For bortezomib treatment, eight-week-old male C57BL/6 mice (Orient Bio Inc.) were fed either an HFD or a chow diet (D12450K; Research Diets Inc.) for 12 weeks, and intraperitoneal injections of bortezomib (0.5 mg/kg) were administered once weekly, starting 5 weeks after HFD initiation (total of 7 weeks). CerS2 heterozygotes or null mice were generated as described previously^25,26^. To induce NAFLD in CerS2 heterozygotes, CerS2 heterozygotes and their littermate WT control mice were fed either an HFD or a chow diet for 12 weeks. For ER stress inhibition, mice were fed an HFD or chow diet with or without 1 g/kg/day of 4-PBA supplemented in the drinking water, as described previously^27^.

### Thin-layer chromatography (TLC)

TLC analysis of neutral lipids (TG, cholesterol, cholesterol ester) was performed, as previously described^7^. Briefly, total lipids were extracted using chloroform/methanol (2:1 by volume) and separated by TLC in heptane/isopropyl ether/acetic acid (60:40:3 by volume). TLC plates were dried, sprayed (15.6% copper sulfate, 9.4% phosphoric acid), and incubated (110 °C, 8–10 min).

### Histology and H&E staining

Hepatic tissues were fixed in 4% paraformaldehyde solution (4 °C) overnight, embedded in paraffin, and then sectioned (4 μm) for H&E staining.

### Real-time polymerase chain reaction (PCR)

Total mRNA of liver or cells was extracted using RNeasy mini kits (Qiagen, Valencia, CA). From extracted mRNA, cDNA was synthesized using a Verso cDNA Synthesis Kit (Thermo Scientific, Hudson, NH). Real-time PCR entailed use of the SYBR Green Real-Time PCR Master Mix (Life Technologies, Grand Island, NY) and an ABI PRISM 7500 Sequence Detection System (Applied Biosystems, Waltham, MA), and relative gene expression was calculated as 2^−ΔΔCt ^^28,29^. The primers used are described in Supplementary Table 1.

### Western blot analysis

Tissues or cells were lysed by radioimmunoprecipitation assay buffer (Tris–Cl [50 mM, pH 7.5], NaCl [150 mM], 1% Nonidet P-40, 0.5% sodium deoxycholate, 0.1% SDS, and protease inhibitors) and homogenized. Proteins in lysates were quantified using Protein Assay Dye Reagent (Bio-Rad Laboratories, Hercules, CA). A protein aliquot (20–50 µg) was loaded in each lane and separated by 8–10% SDS-PAGE and then transferred to nitrocellulose membranes (Bio-Rad Laboratories). Blocking was achieved using 5% bovine serum albumin (BSA) (Sigma-Aldrich, St Louis, MO) in TBST (TBS with 0.1% Tween-20), and the membranes were incubated overnight (4 °C) in primary antibodies in TBST. After washing, secondary antibodies in TBST were added. For chemiluminescence, ECL Western Blotting Detection Reagents (Amersham Biosciences, Little Chalfont Bucks, UK) were used in conjunction with a ChemiDoc MP Imaging System (Bio-Rad Laboratories).

### Cell culture and generation of CerS2 and CerS6-knockdown cells

Hep3B cells were cultured in Dulbecco’s modified Eagle’s medium (DMEM) (HyClone Laboratories, Logan, UT) supplemented with 10% fetal bovine serum (FBS) (HyClone) and 1% penicillin/streptomycin (HyClone). The CerS2 or CerS6 gene was deleted from Hep3B cells with the CRISPR/Cas9 genome editing system and the GeneArt CRISPR nuclease vector with a CD4 enrichment kit (Invitrogen, Carlsbad, CA, USA), as described previously^30^. Briefly, CerS2 target‐specific oligonucleotides (top strand, 5′‐GCCGATCTAGAAGACCGAGAGTTTT‐3′; bottom strand, 5′‐GATCGGTCTTCTAGATCGGTCGGTG‐3′) and CerS6 target-specific oligonucleotides (top strand, 5’-GCTCCCGCACAATGTCACCTGTTTT-3’; bottom strand, 5’-AGGTGACATTGTGCGGGAGCCGGTG‐3′) were designed^31^ and synthesized by Macrogen (Seoul, South Korea). Then, a double‐stranded oligonucleotide with compatible ends was cloned into the GeneArt CRISPR nuclease vector.

### Cell treatment and transfection

To induce ER stress, 500 μM palmitate was added to Hep3B cells for 12 h. In some cases, the cells were preincubated with 4-PBA (5 mM), TUDCA (500 μM) and fumonisin B1 (FB1, 20 μM) for 1 h or 12 h before palmitate treatment. In some cases, 1 μM C16~C24 ceramide pretreatment was used, and cells were permeabilized with 10 μg/ml digitonin, as previously described^7^. Cell transfection was performed using Metafectene (Biontex Laboratories, Munich, Germany) according to the manufacturer’s protocol.

### Triglyceride measurement

Total triglyceride in the liver was measured using colorimetric assays (Triglyceride Quantification Kit, Biovision, Palo Alto, CA).

### Cycloheximide chase assay

The stability of CerS2 and CerS6 was measured according to the previous study^32^. Briefly, after transfection with HA-tagged CerS2 or CerS6 for 48 h, Hep3B cells were incubated with cycloheximide (100 μg/ml) for 24–96 h. Cells were lysed in RIPA buffer, and CerS2 or CerS6 protein levels were examined by Western blotting using anti-HA antibody.

### LC–ESI–MS/MS analysis of ceramides

SL analyses by LC-ESI-MS/MS were conducted, as described previously^33^. Briefly, total lipids were extracted with 10 mg tissue, and extracted samples (10 μL) were injected into an HPLC (Agilent 1200 series, Agilent, CA, USA) and separated through a reversed-phase Kinetex C18 column (2.1 × 50 mm, ID: 2.6 μm) (Phenomenex, St. Louis, MO, USA). The HPLC column effluent was introduced into an API 3200 Triple quadruple mass spectrometer (AB Sciex, Toronto, Canada) and analyzed using electrospray ionization in positive mode. Analyses were performed using electrospray ionization in the positive-ion mode with multiple reaction monitoring to select both parent and characteristic daughter ions specific to each analyte simultaneously from a single injection. Data were acquired using Analyst 1.4.2 software (Applied Biosystems).

### Statistical Analysis

Values are expressed as the mean ± SEM. Student’s *t*-test, one-way analysis of variance (ANOVA), or two-way ANOVA were used for data analysis. All computations relied on Prism software (GraphPad Software Inc., San Diego, CA), with statistical significance set at *p* < 0.05.

## Results

### Ceramide acyl chain lengths are differentially altered depending on the duration of a high-fat diet

**Fig. 1:**
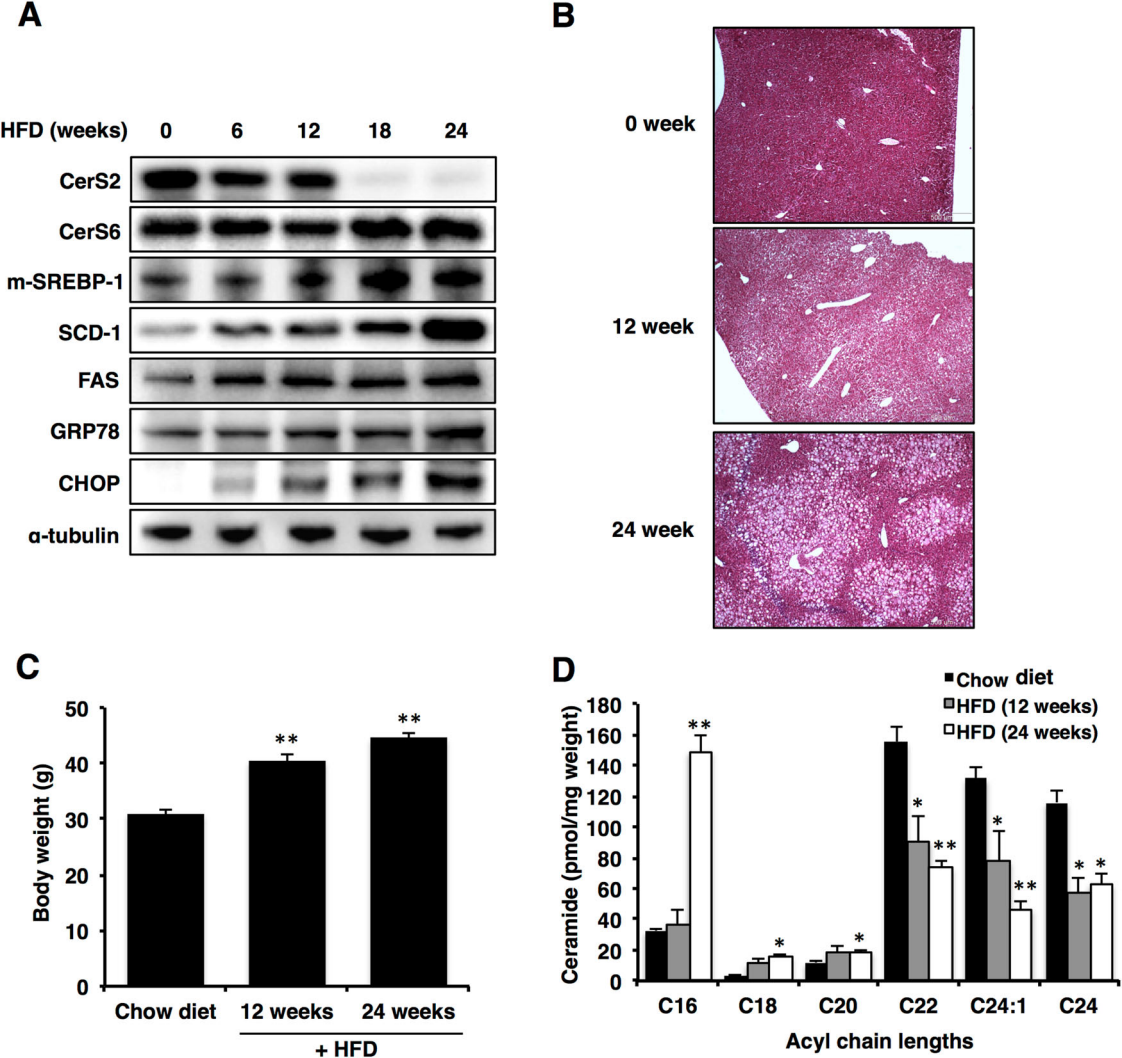
CerS expression is altered during high-fat diet (HFD)-induced nonalcoholic fatty liver disease (NAFLD) progression. **a** Representative Western blots of CerS, the cleaved form of SREBP-1, lipogenic enzymes, and ER stress markers in the liver at several time points (0, 6, 12, 18, and 24 weeks) during HFD administration. **b** Hematoxylin- and eosin-stained liver samples (image magnification, ×40). **c** Body weight in the HFD group compared with the chow diet group. **d** Hepatic ceramide levels during HFD were measured using LC-ESI-MS/MS (*n* = 3). The data are expressed as the means ± SEM; *p < 0.05 and **p < 0.01. Three independent experiments are presented. Abbreviations: CerS, ceramide synthase; CHOP, CCAAT-enhancer-binding protein homologous protein; FAS, fatty acid synthase; GRP78, 78 kDa glucose-regulated protein; HFD, high-fat diet; m-SREBP-1, mature form of sterol regulatory element-binding protein 1; SCD-1, stearoyl-CoA desaturase-1

Increased C16 ceramide levels with a concomitant decrease in C24 ceramide levels in the liver have been reported previously following HFD^23^. To uncover the precise role of each ceramide in the development of NAFLD, we used a diet-induced NAFLD model and examined hepatic CerS expression at several time points (0, 6, 12, 18, and 24 weeks) during HFD (Fig. 1a). The levels of CerS2 were decreased after 6 weeks of HFD (Fig. 1a). In contrast, CerS6 levels were increased after 18 weeks of HFD (Fig. 1a). At the mRNA level, CerS1, CerS5, and CerS6 were increased after 18 weeks of HFD (Supplementary Fig. 1). As reported previously^1^, the expression of ER stress markers (CHOP and GRP78), cleaved SREBP-1, SCD-1, and FAS increased gradually during HFD (Fig. 1a). Hematoxylin and eosin staining revealed vacuolation in hepatocytes, representing steatosis, and the degree of vacuolation was correlated with the duration of HFD administration (Fig. 1b). Body weight was significantly increased at both 12 and 24 weeks of HFD (Fig. 1c). In accordance with CerS levels, the levels of C22-C24 ceramides were decreased at 12 weeks after HFD feeding, while the levels of C16 ceramide were elevated at 24 weeks after HFD feeding (Fig. 1d).

### The effects of ceramide on palmitate-induced ER stress depend on acyl chain length

**Fig. 2:**
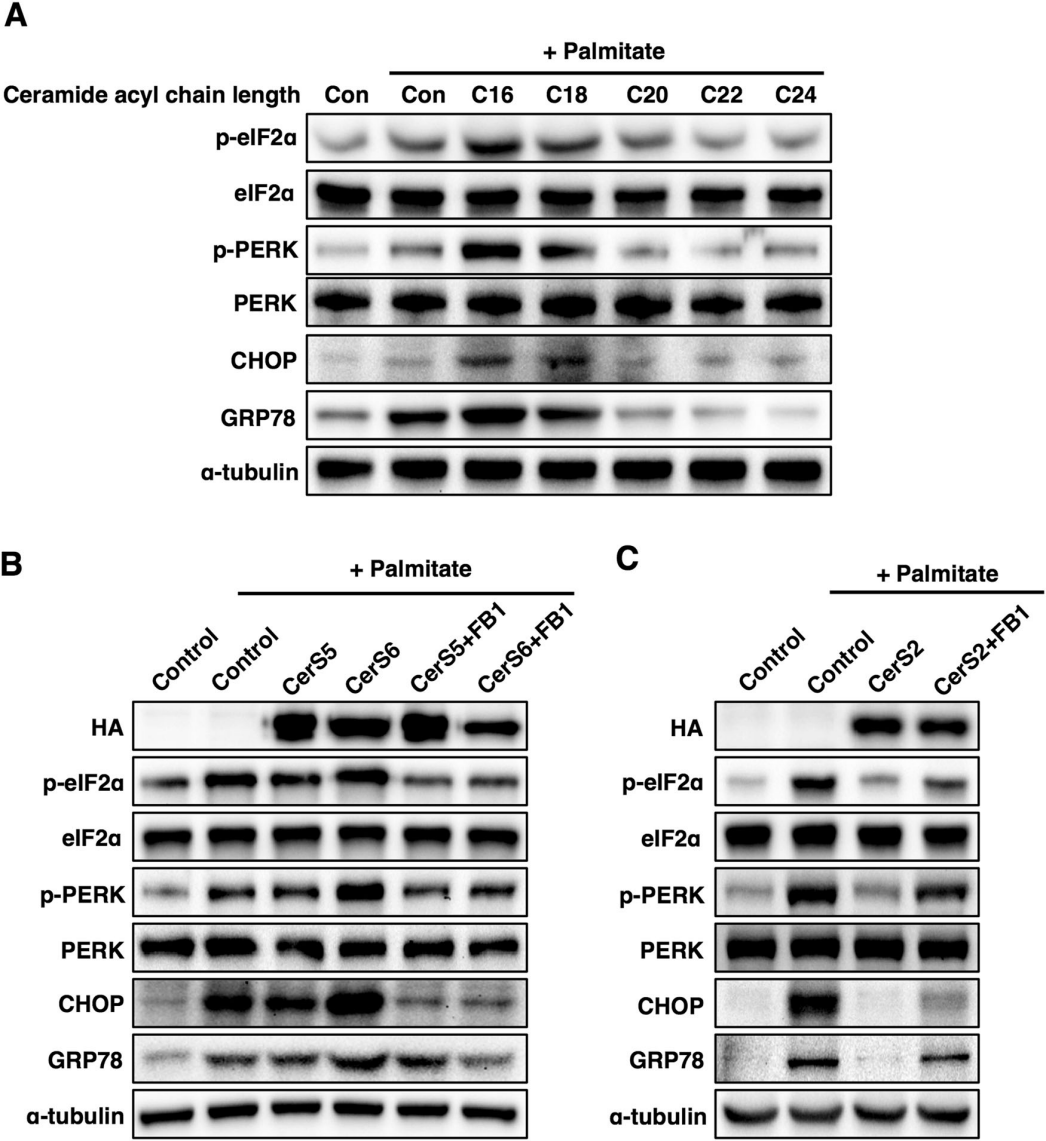
The effects of ceramide on palmitate-induced ER stress are different in Hep3B cells depending on ceramide acyl chain length. **a** Representative Western blots of ER stress markers upon treatment with palmitate (500 μM) and ceramides (1 μM) with various acyl chain lengths. **b** Representative Western blots of ER stress markers upon treatment with palmitate in CerS5- or CerS6-overexpressing cells. **c** Representative Western blots of ER stress markers upon treatment with palmitate in CerS2-overexpressing cells. For CerS inhibition, the cells were pretreated with fumonisin b1 (FB1, 20 µM) 1 h before palmitate treatment. Three independent experiments are presented. *CerS* ceramide synthase, *CHOP* CCAAT-enhancer-binding protein homologous protein, *Con* control, *eIF2α* eukaryotic initiation factor 2α, *GRP78*, 78 kDa glucose-regulated protein, *PERK* Protein kinase RNA-like endoplasmic reticulum kinase

NAFLD is caused by the accumulation of saturated fatty acids in the liver^34^. To clarify the role of ceramide acyl chain length in NAFLD, Hep3B cells (a human liver cell line) were incubated with ceramides containing different acyl chain lengths, and then cells were exposed to the saturated fatty acid palmitate to induce ER stress, as reported previously^34^. C16 and C18 ceramides enhanced palmitate-induced ER stress, while C22-C24 ceramides mitigated palmitate-induced ER stress (Fig. 2a). To confirm the different effects of ceramide with distinct acyl chain lengths, we transfected Hep3B cells with CerS. Similar to incubation with C16 ceramide, CerS6 overexpression enhanced palmitate-induced ER stress, and pretreatment with fumonisin B1 (a ceramide synthase inhibitor^35^) abolished the detrimental effects of CerS6 overexpression on ER stress (Fig. 2b), indicating that the C16 ceramides generated by CerS6 are involved in the phenotype. However, CerS5 overexpression minimally affected palmitate-induced ER stress (Fig. 2b). In contrast, CerS2 overexpression restored palmitate-induced ER stress, and pretreatment with fumonisin B1 abolished the protective effects of CerS2 overexpression on palmitate-induced ER stress (Fig. 2c), suggesting that the C22-C24 ceramides generated by CerS2 can reduce palmitate-induced ER stress.

### CerS6 overexpression increases SREBP-1 cleavage via the reduction of INSIG-1 levels

**Fig. 3:**
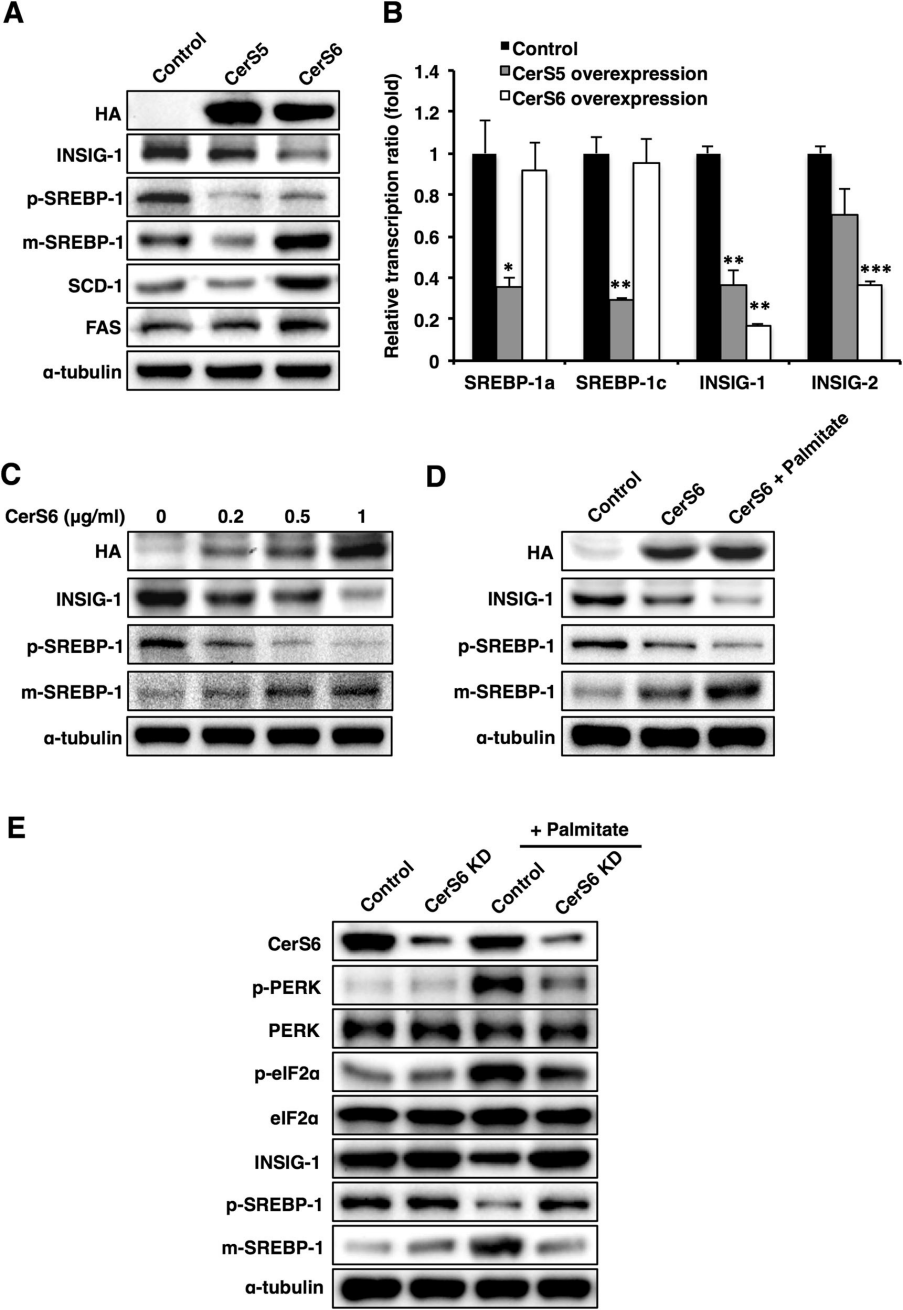
CerS6 expression levels regulate SREBP-1 cleavage and lipogenic enzymes. **a** Representative Western blots of INSIG-1, SREBP-1, lipogenic enzymes, and ER stress markers in CerS5/6-transfected Hep3B cells. **b** Real-time polymerase chain reaction (PCR) of SREBP-1 and INSIG-1/2 in CerS5/6-transfected Hep3B cells. **c** Representative Western blots of INSIG-1 and SREBP-1 in Hep3B cells transfected with CerS6 at different doses (0, 0.2, 0.5, and 1 µg/ml). **d** Representative blots of INSIG-1 and SREBP-1 in CerS6-overexpressing Hep3B cells with or without palmitate treatment. **e** Representative blots of CerS6, ER stress markers, INSIG-1, and SREBP-1 in CerS6-knockdown Hep3B cells with or without palmitate treatment. Three independent experiments are presented. Data are expressed as the means ± SEM *p < 0.05, **p < 0.01, ***p < 0.001. CerS ceramide synthase, CHOP CCAAT-enhancer-binding protein homologous protein, eIF2α eukaryotic initiation factor 2α, GRP78 78 kDa glucose-regulated protein, FAS fatty acid synthase, INSIG Insulin-induced gene 1 protein, m-SREBP-1 mature form of sterol regulatory element-binding protein 1, p-SREBP-1 precursor form of sterol regulatory element-binding protein, 1PERK Protein kinase RNA-like endoplasmic reticulum kinase, SCD-1 stearoyl-CoA desaturase-1

Because the effects of ceramide on palmitate-induced ER stress depend on its acyl chain length, we next explored whether ceramide exerts distinct effects on fatty acid synthesis depending on acyl chain length. Fatty acid synthesis is a pivotal process in the development of NAFLD, and SREBP-1 transcriptionally regulates genes involved in fatty acid synthesis, such as SCD-1 and FAS. In addition, the cleavage and release of SREBP-1 and its subsequent activation are inhibited by insulin-induced gene 1 (INSIG-1)^36^. To directly investigate the roles of C16 ceramide in fatty acid synthesis, SREBP-1 cleavage was examined upon CerS5 or CerS6 overexpression in Hep3B cells. CerS5 overexpression decreased both precursor and mature forms of SREBP-1 protein (Fig. 3a) and decreased mRNA levels of SREBP-1a and SREBP-1c (Fig. 3b), suggesting that CerS5 or the C16 ceramide made by CerS5 suppresses SREBP-1 transcription. Interestingly, CerS6 increased only the mature form of SREBP-1, indicating increased SREBP-1 cleavage with a concomitant increase in SCD-1 and FAS (Fig. 3a). Because CerS6 transfection reduced INSIG-1 levels, leading to increased SREBP-1 cleavage in a dose-dependent manner (Fig. 3c), CerS6-induced SREBP-1 cleavage could be attributed to the decrease in INSIG-1. In addition, palmitate addition aggravated this phenomenon (Fig. 3d). Finally, CerS6 knockdown abrogated palmitate-induced ER stress and SREBP-1 cleavage (Fig. 3e), suggesting a role for CerS6 in SREBP-1 activation and lipogenesis.

**Fig. 4:**
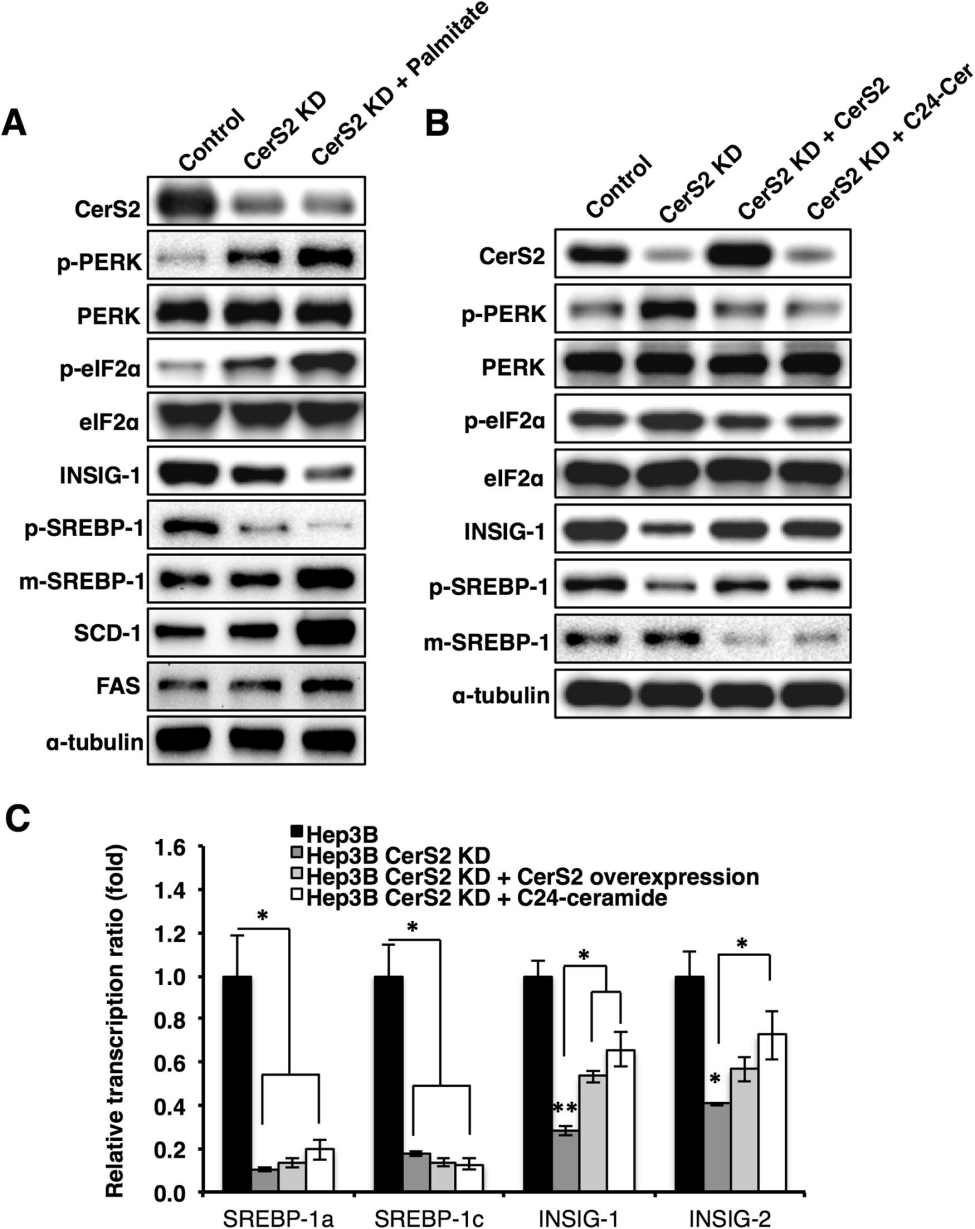
CerS2 knockdown increases the ER stress response in Hep3B cells. **a** Representative Western blots of ER stress and lipogenic pathway components in CerS2-knockdown Hep3B cells with or without palmitate treatment. **b** After CerS2 or C24 ceramide was reintroduced into CerS2-knockdown Hep3B cells, the protein levels of ER stress markers, INSIG-1, and SREBP-1 were examined by Western blotting. **c** The relative SREBP-1 and INSIG-1/2 gene expression levels were analyzed using real-time PCR in CerS2-knockdown Hep3B cells with or without CerS2 reintroduction. The data are expressed as the means ± SEM. *p < 0.05. Three independent experiments are presented. CerS ceramide synthase, FAS fatty acid synthase, INSIG Insulin-induced gene 1 protein, m-SREBP-1 mature form of sterol regulatory element-binding protein 1, p-SREBP-1 precursor form of sterol regulatory element-binding protein 1, SCD-1 stearoyl-CoA desaturase-1

Because HFD reduced the levels of hepatic C22-C24 ceramides (Fig. 1d), we created CerS2-knockdown Hep3B cells using Crispr-Cas9^30^. In contrast to CerS2 overexpression, which mitigated palmitate-induced ER stress (Fig. 2c), CerS2 knockdown caused ER stress and decreased INSIG-1 (Fig. 4a). In addition, palmitate treatment amplified the ER stress response induced by the decrease in CerS2 and increased SREBP-1 cleavage (Fig. 4a). However, despite ER stress induction, CerS2 knockdown did not increase SCD-1 and FAS protein levels without palmitate treatment (Fig. 4a). CerS2 overexpression or C24 ceramide addition in CerS2-knockdown Hep3B cells reversed ER stress and partially recovered the INSIG-1 and SREBP-1 levels (Fig. 4b). Interestingly, CerS2 overexpression or C24 ceramide addition did not recover the mRNA levels of SREBP-1 but partially recovered INSIG-1 mRNA levels (Fig. 4c). Therefore, C22-C24 ceramides do not directly affect SREBP-1 expression but regulate SREBP-1 cleavage via INSIG-1 expression and ER stress modulation.

### SREBP-1 cleavage regulated by CerS is mediated via ER stress

**Fig. 5:**
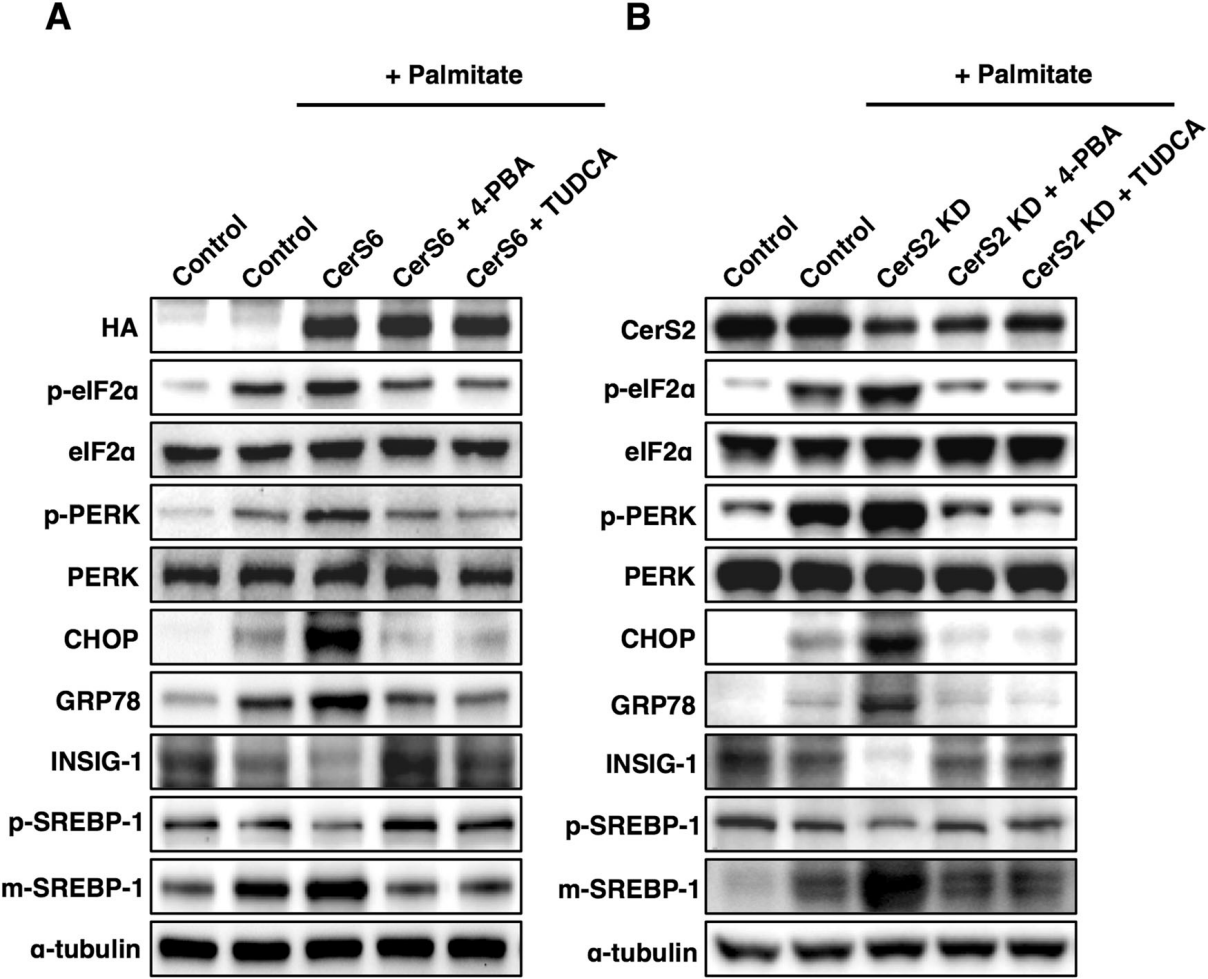
CerS-mediated SREBP-1 cleavage is regulated via ER stress. Representative Western blots of ER stress and lipogenic pathways in **a** CerS6-overexpressing Hep3B cells and **b** CerS2-knockdown Hep3B cells. For ER stress inhibition, the cells were preincubated with 4-PBA (5 mM) and TUDCA (500 μM) for 1 h before palmitate treatment. Three independent experiments are presented. CerS ceramide synthase, CHOP CCAAT-enhancer-binding protein homologous protein, eIF2α eukaryotic initiation factor 2α, GRP78 78 kDa glucose-regulated protein, INSIG Insulin-induced gene 1 protein, KD knockdown, PERK Protein kinase RNA-like endoplasmic reticulum kinase, m-SREBP-1 mature form of sterol regulatory element-binding protein 1, p-SREBP-1 precursor form of sterol regulatory element-binding protein 1

To explore whether the ER stress response induced by CerS modulation was involved in SREBP-1 cleavage, we pretreated Hep3B cells with ER stress inhibitors such as 4-PBA and TUDCA. ER stress inhibition abrogated the decrease in INSIG-1 and increased SREBP-1 cleavage induced by CerS6 overexpression (Fig. 5a) or CerS2 knockdown (Fig. 5b). These results indicate that ER stress is upstream of the SREBP-1 cleavage modulated by CerS.

### CerS2 knockdown in vivo aggravates NAFLD progression via a more robust ER stress response and increased SREBP-1 cleavage

**Fig. 6:**
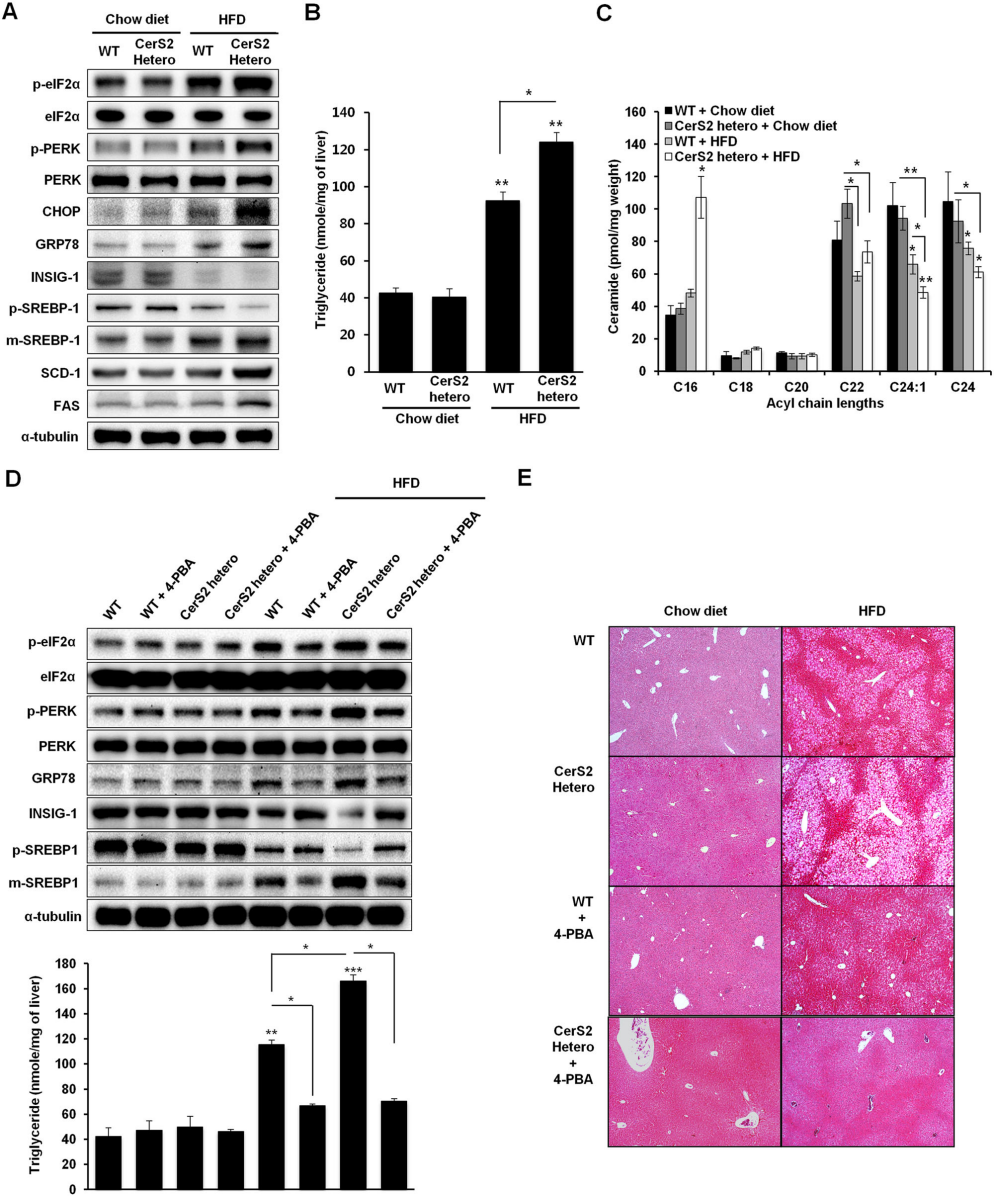
CerS2 haploinsufficiency exacerbates steatosis via a stronger ER stress response and increased SREBP-1 cleavage in vivo. **a** Representative Western blots of ER stress and lipogenic pathway components following the administration of the chow diet and HFD. **b** Hepatic TG levels quantified by a commercial colorimetric kit. **c** Hepatic ceramide levels were measured using LC-ESI-MS/MS (*n* = 3). To inhibit ER stress in vivo, mice were fed an HFD or a chow diet with or without 1 g/kg/day 4-PBA supplemented in the drinking water. **d** Representative Western blots of ER stress and lipogenic pathway components are shown, and the results of hepatic TG levels quantified by a commercial colorimetric kit are shown. **e** Hematoxylin- and eosin-stained liver samples (image magnification, ×40) following chow diet and HFD administration with or without ER stress inhibition. The data are expressed as the means ± SEM. *p < 0.05, **p < 0.01, and ***p < 0.001. Three independent experiments are presented. CerS ceramide synthase, CHOP CCAAT-enhancer-binding protein homologous protein, eIF2α eukaryotic initiation factor 2α, FAS fatty acid synthase, GRP78 78 kDa glucose-regulated protein, Hetero heterozygote, HFD high-fat diet; INSIG, Insulin-induced gene 1 protein, PERK Protein kinase RNA-like endoplasmic reticulum kinase, m-SREBP-1 mature form of sterol regulatory element-binding protein 1, p-SREBP-1 precursor form of sterol regulatory element-binding protein 1, SCD-1 stearoyl-CoA desaturase-1

To confirm the protective role of CerS2 in NAFLD, we induced NAFLD in CerS2 heterozygote mice^22^ by administering an HFD for 12 weeks. Because CerS2 null livers display CD36 mislocalization and insulin receptor dysfunction with altered detergent-resistant membranes^7,21^, CerS2 null livers did not develop NAFLD during HFD^7^ despite an increased hepatic ER stress response (Supplementary Fig. 2). In addition, the lack of iNKT cells in CerS2-null mice^37^ could also affect NAFLD progression^38^. Thus, CerS2 heterozygotes were used to induce NAFLD to exclude other factors that are altered in CerS2 null mice. CerS2 heterozygotes have been reported to confer susceptibility to diet-induced steatohepatitis and insulin resistance^22^. However, the present study demonstrates the relationship between CerS2 haploinsufficiency and ER stress that is linked to steatosis. In the groups fed the chow diet, CerS2 heterozygotes showed no difference from WT littermates in ER stress response and SREBP-1 cleavage (Fig. 6a). However, hepatic ER stress response and SREBP-1 cleavage were significantly enhanced in CerS2 heterozygotes upon HFD administration (Fig. 6a). In the livers of HFD-fed CerS2 heterozygotes, the expression levels of SCD-1 and FAS, as well as lipid accumulation, as demonstrated by vacuolation in hepatocytes, were also increased in accordance with elevated SREBP-1 cleavage (Fig. 6a, Supplementary Fig. 3). Hepatic TG levels were also significantly increased in CerS2 heterozygotes compared with WT littermates upon HFD administration (Fig. 6b). Although significant alterations in the ceramide acyl chain length were not observed in CerS2 heterozygote livers under the chow diet, the increase in C16 ceramide and the decrease in C24:1 ceramide were significant upon HFD administration in CerS2 heterozygotes compared with those in WT littermates (Fig. 6c), implicating that CerS2 haploinsufficiency leads to increased hepatic lipogenesis and a stronger ER stress response by altering ceramide acyl chain length. Moreover, the ER stress inhibition induced in CerS2 heterozygote mice by feeding 4-PBA reversed the enhanced ER stress response and SREBP-1 cleavage in the HFD group (Fig. 6d). In addition, the HFD-induced increase in hepatic TG levels (Fig. 6d) and vacuolation in hepatocytes (Fig. 6e) were also normalized with ER stress inhibition in CerS2 heterozygotes, indicating that ER stress is upstream of the SREBP-1 cleavage modulated by CerS2 and plays a critical role in aggravating NAFLD progression in CerS2 heterozygotes.

### Increased CerS2 levels in vivo protect the liver from NAFLD progression

**Fig. 7:**
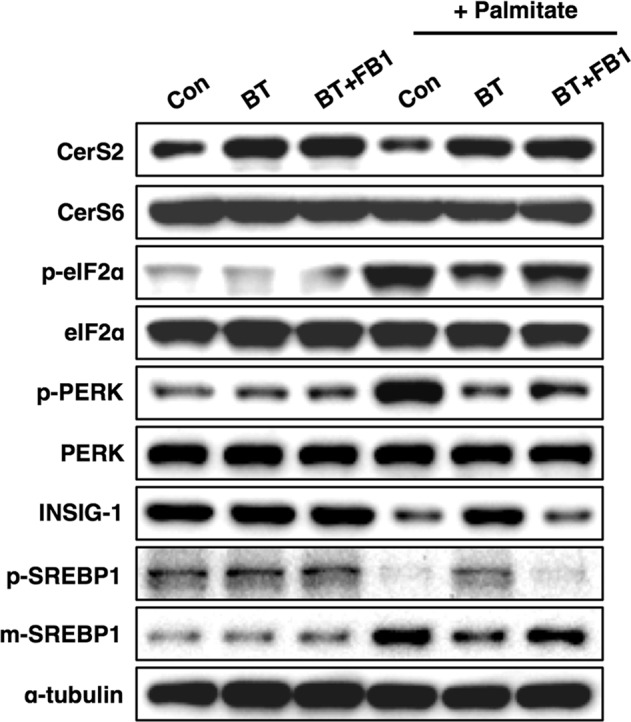
Bortezomib-induced increase in CerS2 alleviates palmitate-induced ER stress. The cells were pretreated with bortezomib (BT, 50 nM) and fumonisin B1 (FB1, 20 µM) 12 h before palmitate (500 μM) treatment. After 12 h of palmitate treatment, CerS, ER stress markers, SREBP-1, and INSIG-1 protein levels were examined using Western blotting. The images are representative images of three independent experiments. CerS ceramide synthase, Con control, BT Bortezomib, eIF2α eukaryotic initiation factor 2α, INSIG-1 Insulin-induced gene 1 protein, m-SREBP-1 mature form of sterol regulatory element-binding protein 1, p-SREBP-1 precursor form of sterol regulatory element-binding protein 1, PERK Protein kinase RNA-like endoplasmic reticulum kinase

**Fig. 8:**
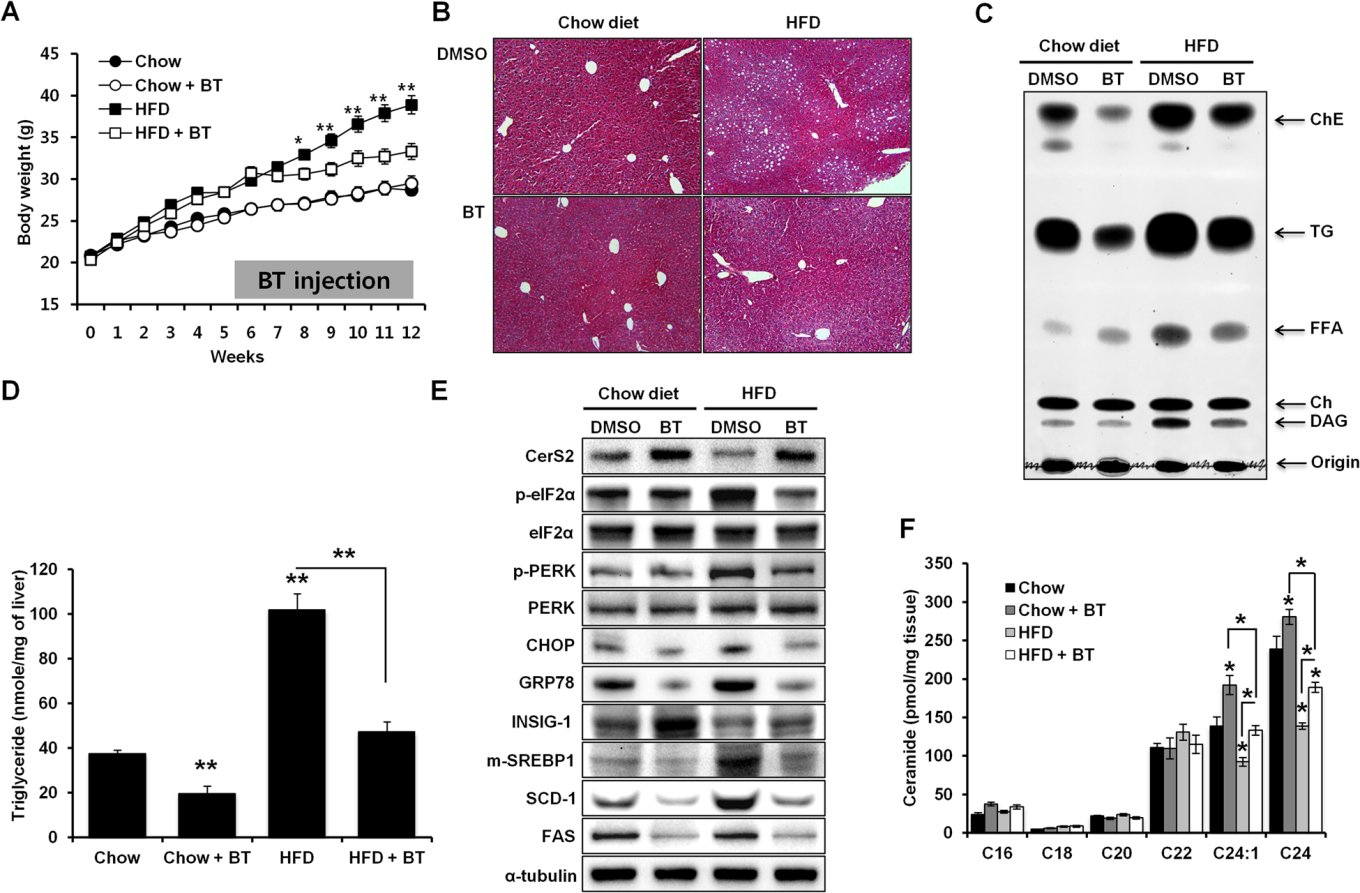
Increase in CerS2 induced by bortezomib injections alleviates steatosis following a high-fat diet (HFD). **a** Weight gain in mice (*n* = 8) fed an HFD for 5 weeks, followed by bortezomib injections (0.5 mg/kg/week) for 7 weeks. **b** Hematoxylin- and eosin-stained liver samples (image magnification, ×40). **c** Hepatic TG and cholesterol ester levels determined by thin-layer chromatography. **d** Hepatic TG levels quantified by a commercial colorimetric kit. **e** Representative Western blots of CerS2, ER stress, and lipogenic pathway components in the liver upon bortezomib injection. **f** Hepatic ceramide levels were measured using LC-ESI-MS/MS (*n* = 3). The data are expressed as the means ± SEM. *p < 0.05, **p < 0.01. Three independent experiments are presented. BT bortezomib, CerS ceramide synthase, Ch cholesterol, ChE cholesterol ester, CHOP CCAAT-enhancer-binding protein homologous protein, DAG diacylglycerol, eIF2α eukaryotic initiation factor 2α, FAS fatty acid synthase, FFA free fatty acid, GRP78 78 kDa glucose-regulated protein, Hetero heterozygote, HFD high-fat diet, INSIG Insulin-induced gene 1 protein, PERK Protein kinase RNA-like endoplasmic reticulum kinase, m-SREBP-1 mature form of sterol regulatory element-binding protein 1, p-SREBP-1 precursor form of sterol regulatory element-binding protein 1, SCD-1 stearoyl-CoA desaturase-1, TG triglyceride

Next, we explored whether increased CerS2 levels play a protective role in NAFLD progression in vivo. To identify drugs that can modulate CerS expression, we searched various candidates, including proteasome and lysosome inhibitors, and found that bortezomib treatment in Hep3B cells can specifically increase CerS2 protein levels (Fig. 7). In addition, bortezomib treatment in Hep3B cells reversed palmitate-induced ER stress and SREBP-1 cleavage with a concomitant increase in CerS2 (Fig. 7). Bortezomib administration at a low dosage has also been reported to play a protective role in alcoholic fatty liver disease^39^. Thus, to determine whether bortezomib administration might alleviate NAFLD and improve triglyceride (TG) metabolism in vivo, mice were first given HFD for 5 weeks to initiate steatosis^40^, along with once weekly intraperitoneal injections of bortezomib (0.5 mg/kg/week) for 7 weeks, while the chow diet or HFD was maintained. The weight gain was lower upon bortezomib treatment (Fig. 8a), and hematoxylin- and eosin (H&E)-stained liver sections showed that lipid droplets decreased upon bortezomib treatment (Fig. 8b). Lower TG levels upon bortezomib injection were confirmed by thin-layer chromatography and a colorimetric assay kit (Fig. 8c, d). In accordance with the Hep3B cell data (Fig. 7), bortezomib injection increased hepatic CerS2 expression and alleviated the HFD-induced ER stress response with concomitant reduced SREBP-1 cleavage, SCD-1, and FAS (Fig. 8e). In accordance with increased hepatic CerS2 by bortezomib injections, bortezomib administration elevated C24:1 and C24 ceramides in the liver (Fig. 8f). In addition, bortezomib recovered the decreased levels of C24:1 and C24 ceramides upon HFD (Fig. 8f).

Finally, the degradation rates of CerS2 and CerS6 were examined by inhibiting protein synthesis with cycloheximide in Hep3B cells (Supplementary Fig. 4a) to uncover the mechanism by which bortezomib treatment only affects CerS2, but not CerS6, expression. The degradation rate of CerS2 was faster than that of CerS6 (Supplementary Fig. 4a), implicating the rapid turnover of CerS2. Both CerS2 and CerS6 can be ubiquitinated (data not shown). Possibly due to the fast turnover rate of CerS2, CerS2 protein levels were greatly increased upon proteasome inhibition. To elucidate whether CerS6 protein levels are also elevated upon the proteasome inhibition induced by the higher dosage, we administered different doses of bortezomib and examined the protein levels of CerS2 and CerS6 (Supplementary Fig. 4b). While CerS2 protein levels were elevated upon proteasome inhibition as expected, CerS6 protein levels were further decreased upon proteasome inhibition with doses higher than 0.5 µM (Supplementary Fig. 4b). Lysosome inhibition using chloroquine did not affect either CerS2 or CerS6 protein levels (Supplementary Fig. 4b).

## Discussion

NAFLD represents hepatic fatty changes (steatosis) in the absence of a history of excessive alcohol consumption and is usually accompanied by features of metabolic disorders such as insulin resistance^2^. The spectrum of NAFLD histologically spans from generally benign, bland steatosis to steatosis with evidence of hepatocellular inflammation and damage (nonalcoholic steatohepatitis, or NASH), which may be complicated by progressive fibrosis and cirrhosis^2^. Ceramides have been highlighted as major contributors to the tissue dysfunction underlying metabolic pathologies, and the distinct roles of ceramides with specific acyl chain lengths have been recently revealed via studies with CerS-deficient mice^12^. The present study demonstrates that ceramides with different acyl chain lengths, generated by CerS2 and CerS6, modulate the ER stress response and SREBP-1 cleavage, which eventually affects hepatic lipogenesis and NAFLD progression.

In the present study, we explored the roles of CerS2, CerS5, and CeS6 in NAFLD progression. CerS2 mainly produces C22-C24 ceramides (very long-chain ceramides)^14^, and CerS5 and CerS6 generate C16 ceramide (long-chain ceramide)^17,18^. The effects of ceramides on palmitate-induced ER stress were different depending on acyl chain lengths. Whereas treatment with long-chain ceramide exacerbated the palmitate-induced ER stress response, the effects of very long-chain ceramides on palmitate-induced ER stress were protective. Several previous studies have investigated ceramide-induced ER stress^41,42^. For example, C2 ceramide treatment induced cancer cell death through a mechanism involving severe ER stress induced by the disruption of ER calcium homeostasis^41^. In addition, CerS6 downregulation was observed in ER stress induced by tunicamycin or suberoylanilide hydroxamic acid, and the knockdown of CerS6 was involved in ATF-6 activation and ER stress-mediated apoptosis in head and neck squamous cell carcinoma (HNSCC) cell lines^42^. The detrimental effects of CerS6 and C16 ceramide on palmitate-induced ER stress demonstrated in the present study are contrary to the previously reported effects of CerS6 on tunicamycin-induced ER stress^42^. Whereas CerS6 downregulation in HNSCC cells was observed upon tunicamycin-induced ER stress^42^, CerS6 upregulation was observed in fatty liver with a concomitant ER stress response during HFD administration, and CerS6 overexpression in Hep3B cells exacerbated ER stress markers under palmitate-induced ER stress. In addition, CerS6 knockdown in Hep3B cells relieved the ER stress response not only in palmitate-induced ER stress but also in thapsigargin-induced ER stress, and CerS2 knockdown showed the opposite effects (Supplementary Fig. 5). The different results may be derived from the difference in cell characteristics or ER stress inducers.

Different effects of ceramides depending on acyl chain lengths have been reported previously. For example, CerS1/C18 ceramides suppress HNSCC xenograft tumor growth, and CerS6/C16 ceramides induce HNSCC tumor proliferation in SCID mice^43^. In addition, CerS2/C22-C24 ceramides partially alleviate irradiation-induced apoptosis, whereas CerS5/C16 ceramides induce HeLa cell apoptosis^44^. Similarly, an altered SL composition from C24 to C16 increases susceptibility to apoptosis in HeLa cells^45^, and long-chain ceramides and very long-chain ceramides have opposite effects on human breast and colon cancer cell growth^46^. Thus, the balance between very long- and long-chain ceramides has emerged as a novel mechanism in the regulation of cell death^12,46–48^. In accordance with previous reports, treatment with C22-C24 ceramides and C16 ceramide, as well as the overexpression of CerS2 and CerS5/6, showed opposite effects under palmitate-induced ER stress. Altered membrane properties by changing the SL acyl chain composition have been reported^21,26^, and membrane composition and properties are important for the ER stress response. For example, the modulation of membrane phospholipid composition by liver X receptors regulates ER stress^49^, and the membrane expansion driven by lipid biosynthesis alleviates ER stress independently of the unfolded protein response^50^. The mechanism underlying how ceramides with different acyl chain lengths exhibit opposite effects on palmitate-induced ER stress should be further elucidated.

CerS6 overexpression and CerS2 knockdown decreased INSIG-1 expression, as well as increased SREBP-1 cleavage, leading to higher lipogenesis, and ER stress played a role in CerS-regulated SREBP-1 activation. The involvement of ER stress in hepatic lipogenesis has been well documented^51^. ER stress can activate SREBPs, leading to the upregulation of genes such as FAS, SCD-1, and acetyl-coenzyme A carboxylase, and the expression of lipogenic enzymes and transcription factors influences hepatic cholesterol and TG biosynthesis, as well as the development of steatosis^51^. CerS2 heterozygotes have already been reported to confer susceptibility to diet-induced steatohepatitis and insulin resistance^22^. Raichur et al. suggested that hepatic TG accumulation in CerS2 heterozygotes is likely due to the impaired lipid oxidation resulting from C16-SL-induced defects in the electron chain^22^. However, the present study, for the first time, demonstrated that the relationship between CerS2 haploinsufficiency and ER stress is linked to steatosis. ER stress has also been reported to inhibit hepatic fatty acid oxidation, which contributed to the early development of hepatic steatosis . Therefore, it is also possible that the higher ER stress response induced in CerS2 heterozygotes contributed to impaired fatty acid oxidation. While the previous study^22,52^ focused on lipid oxidation and very low-density lipoprotein secretion, the present study scrutinized the fatty acid synthetic pathway, including INSIG-1 and SREBP-1, linked to ER stress in CerS2 heterozygotes.

The present study also suggested bortezomib as a CerS2 modulator and a novel treatment for ER stress induced via C16-ceramide accumulation or altered SL ratios. The ubiquitin-proteasome pathway is primarily responsible for the degradation and reuse of cellular proteins that are involved in various intracellular functions, such as signal transduction, cell cycle control, transcriptional regulation, and cell death^53^. In particular, the elimination of misfolded and damaged proteins via the ubiquitin-proteasome pathway is essential for maintaining intracellular homeostasis, and proteasome inhibition usually induces ER stress. However, low-dose bortezomib treatment alleviated diet-induced steatosis and ER stress with a concomitant CerS2 increase. CerS2 protein levels, but not mRNA levels, were decreased during HFD administration, indicating that another mechanism, such as protein degradation, rather than transcriptional regulation, is involved in the HFD-induced decrease in CerS2. CerS2 can be ubiquitinated (data not shown) and may be degraded through the proteasome pathway; thus, proteasome inhibition may enhance CerS2 protein levels. In addition, the protein turnover of CerS2 was faster than that of CerS6, and the difference in protein turnover rates can affect susceptibility to proteasome inhibition. Interestingly, CerS6 protein levels were decreased with high-dose proteasome inhibition. Since proteasome inhibition can increase autophagic degradation^54^ or calpain activity^55^, and bortezomib treatment did not alter the mRNA levels of CerS (data not shown), CerS6 may be degraded through other protein degradation pathways that are activated upon proteasome inhibition. The proteasome inhibitors bortezomib (Velcade®) and carfilzomib (Kyprolis®) were recently approved by the U.S. Food and Drug Administration as therapeutic agents for multiple myeloma and mantle cell lymphoma^53,56,57^. The proteasome pathway has been the object of considerable attention as a target of novel drug development. In the present study, in the increase in CerS2 upon bortezomib administration lowered the levels of cleaved SREBP-1, SCD-1, and FAS, which are important proteins in fatty acid synthesis. SREBP-1a and SREBP-1c are transcription factors that regulate fatty acid synthesis^58^, and the effects of various SLs on SREBP levels have been documented. In particular, C2 ceramide reduces the levels of SREBP-1, SREBP-2^59^, and other SLs, such as lactosylceramide, globoside, GM1, and sphingomyelin, and increases cholesterol levels by promoting the cleavage of SREBP-1^60^. In this study, we demonstrated that SREBP-1 levels are differentially regulated depending on the acyl chain length of ceramide generated by CerS2, CerS5, and CerS6.

Although the modulation of SLs has emerged as a therapeutic target for various diseases, a specific CerS inhibitor has not been reported. Low-dose bortezomib treatment reduced TG accumulation on the chow diet group and prevented HFD-induced obesity and fatty liver development in mice by enhancing CerS2 expression, which plays a key role in regulating ER stress and its downstream targets, SREBP-1 cleavage, and lipogenesis. Proteasome inhibitors are commercially available at present and may be suitable not only to treat obesity and fatty liver disease but also for CerS regulation. In this context, the therapeutic potential of bortezomib may appear to reside in its regulation of CerS2. The involvement of CerS in ER stress may be applied as a therapeutic target for various diseases, considering the important role of ER stress in human diseases.

## Supplementary information

supplementary data
