# Supplementary material for: Hepatic triglyceride accumulation via endoplasmic reticulum stress-induced SREBP-1 activation is regulated by ceramide synthases
Source: Exp Mol Med. 2019 Nov 1;51(11):129. doi: 10.1038/s12276-019-0340-1 (PMC6825147; doi:10.1038/s12276-019-0340-1)
Supplement: Supplementary file 1 — supplementary data [file 12276_2019_340_MOESM1_ESM.docx]

*Electronic supplementary data submitted to Experimental and Molecular Medicine*

**Ceramide synthases modulate hepatic triglycerides via regulating endoplasmic reticulum stress-induced SREBP-1 activation**

Ye-Ryung Kim^1*^, Eun-Ji Lee^1, 2*^, Kyong-Oh Shin^3^, Min Hee Kim^2^,

Yael Pewzner-Jung^4^, Yong-Moon Lee^3^, Joo-Won Park^1#^,

Anthony H. Futerman^4^, and Woo-Jae Park^2,5#^

**^1^**Department of Biochemistry, College of Medicine, Ewha Womans University, Seoul 07084, Republic of Korea; **^2^**Department of Biochemistry, College of Medicine, Gachon University, Incheon 21999, Republic of Korea; **^3^**College of Pharmacy, Chungbuk National University, Chongju 28644, Republic of Korea; **^4^**Department of Biomolecular Sciences, Weizmann Institute of Science, Rehovot 76100, Israel; **^5^**Department of Health Sciences and Technology, GAIHST, Gachon University, Incheon 21999.

^*^Contributed equally to this manuscript

^#^Corresponding Author

**Woo-Jae Park** at Department of Biochemistry, College of Medicine, Gachon University, Gaetbeolro 155, Incheon 21999, Republic of Korea. Tel, +82-32-899-6417; Fax, +82-32-899-4744; e-mail, [ooze@gachon.ac.kr](mailto:ooze@gachon.ac.kr)

**Joo-Won Park** at Department of Biochemistry, College of Medicine, Ewha Womans University, Seoul 07084, Republic of Korea. Tel, +82-2-6986-6201; Fax, +82-2-6986-7016; e-mail, [joowon.park@ewha.ac.kr](mailto:joowon.park@ewha.ac.kr)

Supplementary Table 1. Primers used for real time PCR.

| Gene | Primer sequence (5’-3’) | Reference |
| --- | --- | --- |
| SREBP-1a  (for human) | F: TCAGCGAGGCGGCTTTGGAGCAG | ^1^ |
|  | R: CATGTCTTCGATGTCGGTCAG |  |
| SREBP-1c  (for human) | F: GGAGGGGTAGGGCCAACGGCCT | ^1^ |
|  | R: CATGTCTTCGAAAGTGCAATCC |  |
| INSIG-1  (for human) | F: CATTAACCACGCCAGTGCTA |  |
|  | R: CGATCAAATGTCCACCAAAG |  |
| INSIG-2  (for human) | F: TCCAGTGTAATGCGGTGTGT |  |
|  | R: GCAGCCAGTGTGAGAGACAA |  |
| SREBP-1a  (for mouse) | F: TAGTCCGAAGCCGGGTGGGCGCCGGCGCCAT | ^1^ |
|  | R: GATGTCGTTCAAAACCGCTGTGTGTCCAGTTC |  |
| SREBP-1c  (for mouse) | F: ATCGGCGCGGAAGCTGTCGGGGTAGCGTC | ^1^ |
|  | R: ACTGTCTTGGTTGTTGATGAGCTGGAGCAT |  |
| INSIG-1  (for mouse) | F: CTGTATTGCCGTGTTCGTTG |  |
|  | R: ACCCAAAGAGAGGGCTGCTA |  |
| INSIG-2  (for mouse) | F: CGGGGTGGTACTCTTCTTCA |  |
|  | R: CGTGATCACATCTGGTGGAA |  |

**Supplementary Figures**

**Supplementary Fig. 1** The expression of ceramide synthase (CerS) genes was determined using real-time PCR. Results are means ± S.E.M. (*n* = 4). **P* < 0.05 vs control. HFD, high fat diet


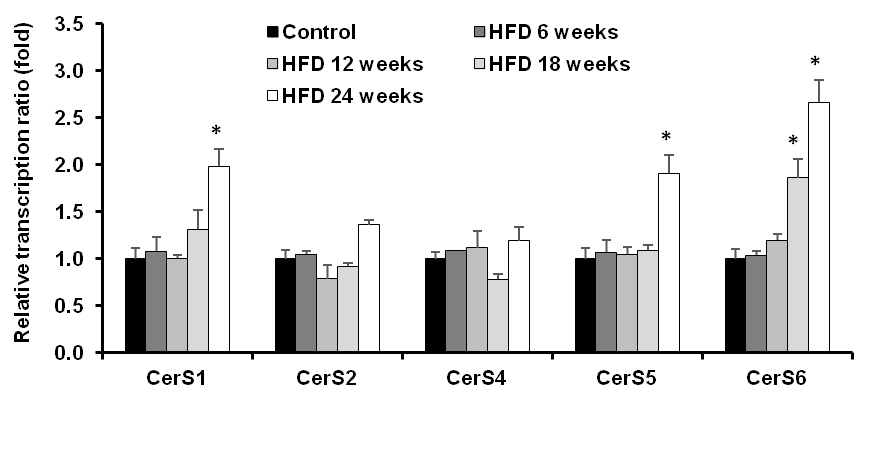


**Supplementary Fig. 2** Increased ER stress in CerS2 null liver. (a) Western blots of ER stress markers, SREBP-1, and INSIG-1. The images are representative images of three independent experiments. (b) Real-time PCR of SREBP-1 and INSIG genes. CHOP, CCAAT-enhancer-binding protein homologous protein; Con, control; eIF2α, eukaryotic initiation factor 2α; Hetero, heterozygote; INSIG-1, Insulin-induced gene 1 protein; m-SREBP-1, mature form of sterol regulatory element-binding protein 1; p-SREBP-1, precursor form of sterol regulatory element-binding protein 1; PERK, Protein kinase RNA-like endoplasmic reticulum kinase. Values are means ± S.E.M. (*n* = 4). **P* < 0.05, ***P* < 0.01 *versus* WT


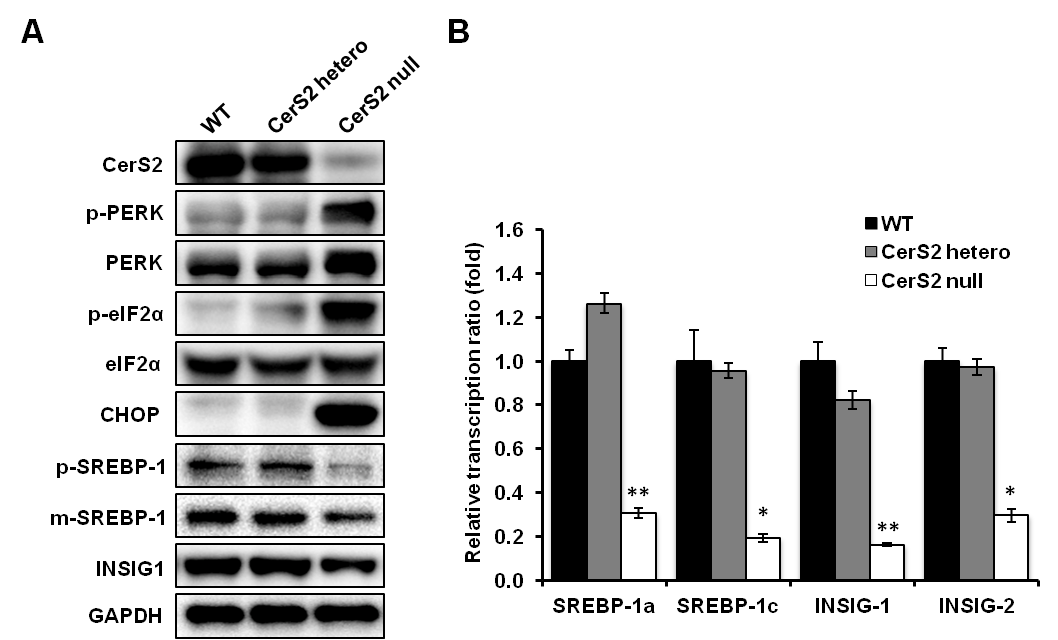


**Supplementary Fig. 3** CerS2 haploinsufficiency exacerbates steatosis. Haematoxylin- & eosin-stained liver samples (image magnification, 40×) following a chow diet and HFD


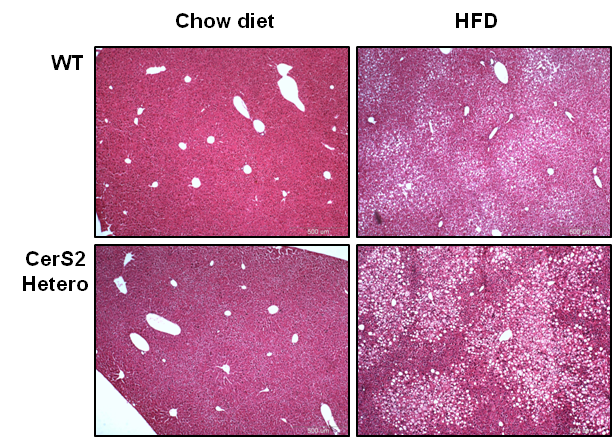


**Supplementary Fig. 4** Faster turnover of CerS2 compared with CerS6. Representative Western blots of CerS2 and CerS6 were shown upon cycloheximide (100 μg/ml) treatment for indicated time (a), and upon bortezomib or chloroquine treatment with different dosages (b) in Hep3B cells. The images are representatives of three independent experiments. CerS, ceramide synthase.

**
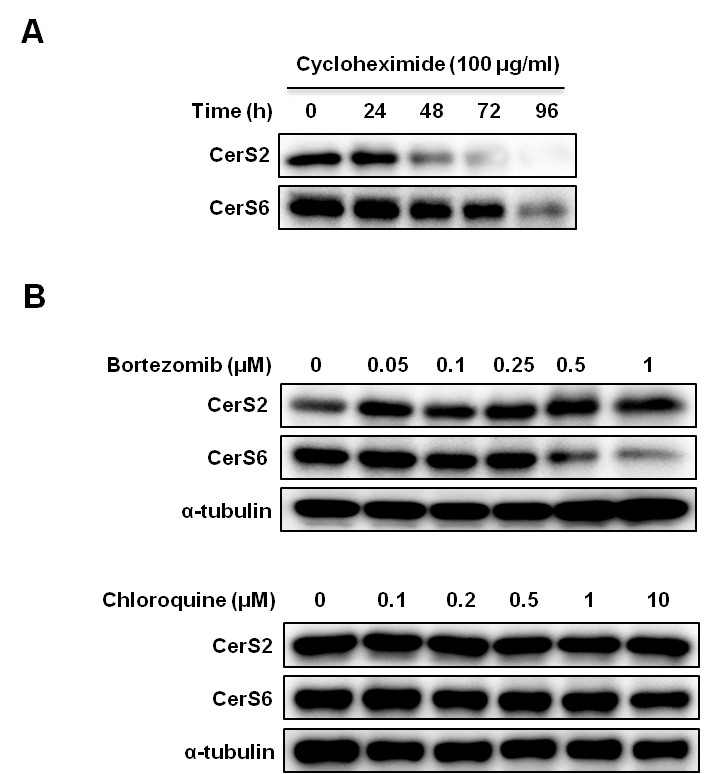
**

**Supplementary Fig. 5** ER stress response was altered in CerS2 or CerS6 knockdown Hep3B cells upon tharpsigargin treatment. Representative Western blots of CerS and ER stress markers were shown upon tharpsigargin (300 nM) treatment for indicated time (a) in CerS6 knockdown Hep3B cells, or (b) in CerS2 knockdown Hep3B cells. CerS, ceramide synthase; CHOP, CCAAT-enhancer-binding protein homologous protein; Con, control; eIF2α, eukaryotic initiation factor 2α; GRP78, 78 kDa glucose-regulated protein; KD, knockdown; KD, knockdown; PERK, Protein kinase RNA-like endoplasmic reticulum kinase. The images are representative images of three independent experiments


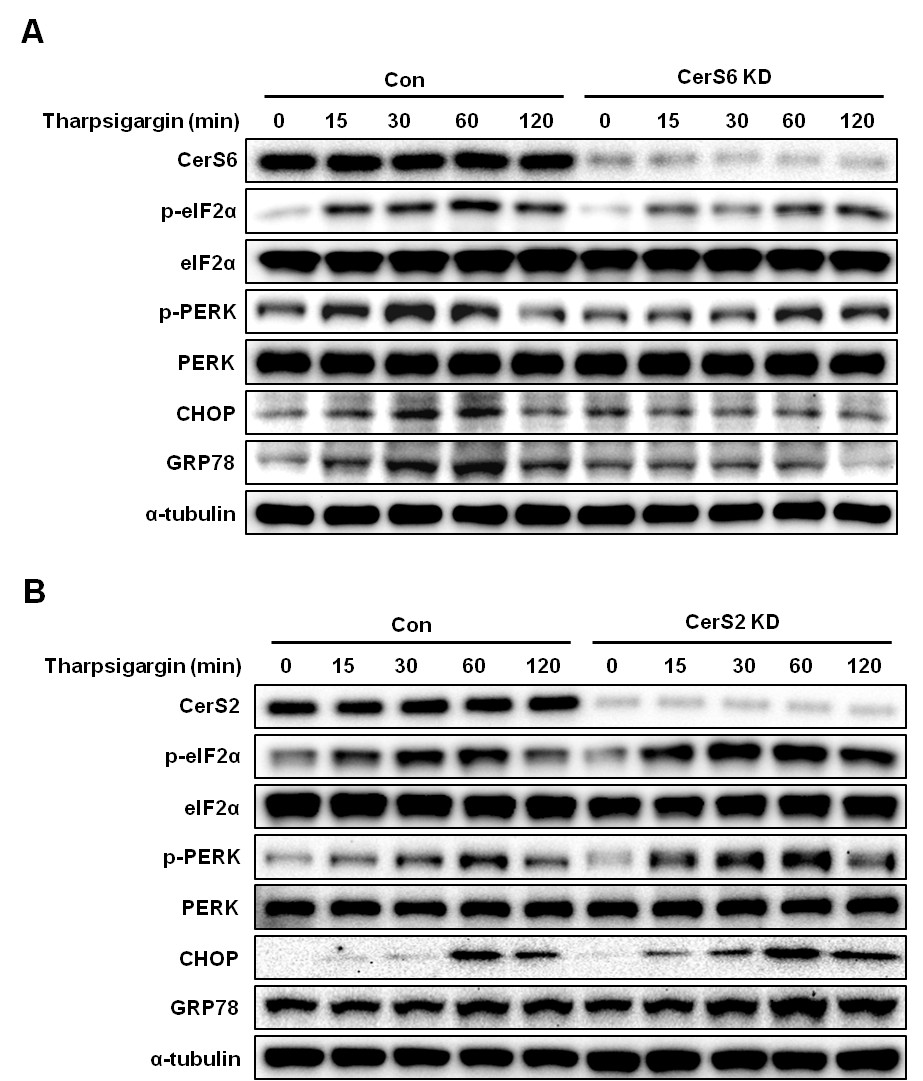


**Reference**

1 Shimomura, I., Shimano, H., Horton, J.D., Goldstein, J.L. & Brown, M.S. Differential expression of exons 1a and 1c in mRNAs for sterol regulatory element binding protein-1 in human and mouse organs and cultured cells. *J. Clin. Invest.* **99**, 838–845 (1997).
